# P20 A case of MDR *Salmonella* Stanleyville bacteraemia in an infant following travel to Nigeria

**DOI:** 10.1093/jacamr/dlaf230.027

**Published:** 2025-12-04

## Abstract

**Background and Objectives:**

*Salmonella* is a leading cause of bacterial foodborne gastrointestinal illness worldwide. Growing antimicrobial resistance (AMR) among travel associated gastroenteritis is a global concern. Sporadic cases of *Salmonella* Stanleyville had been reported earlier in the UK. However, to our knowledge, this is the first report of *Salmonella* Stanleyville bacteraemia in the UK.

**Case summary:**

We describe a 12-month-old infant presenting with fever and lethargy along with 10 days of unresolving diarrhoea after returning from a family visit to Nigeria. She was started on initial supportive treatment and IV ceftriaxone as standard hospital antibiotic policy. However, she remained febrile with rising CRP following 48 h of treatment. Antibiotic was changed to meropenem following a growth of MDR *Salmonella* sp., later identified as *Salmonella* Stanleyville from the admission blood and stool cultures. The same organism was also isolated from a repeat blood culture after 48 h of admission confirming persistent bacteraemia. She recovered after 10 days of hospitalization with broad-spectrum antibiotic treatment.

**Conclusions:**

Clinicians should be vigilant about the choice of antibiotic treatment for travel associated diarrhoea with prolonged fever from west and central African regions where resistance among food-borne pathogens is rapidly rising.

